# Working memory and lexical ambiguity resolution in Cantonese Chinese

**DOI:** 10.1371/journal.pone.0248170

**Published:** 2021-03-10

**Authors:** Michael C. W. Yip

**Affiliations:** Department of Psychology, The Education University of Hong Kong, HKSAR, Hong Kong; University of Central Florida, UNITED STATES

## Abstract

The present study examined how working memory functions in the underlying mechanism of the lexical disambiguation process (in activation approach or in inhibition approach). We recruited sixty native Cantonese listeners to participate in two experimental tasks: (a) a Cantonese-version reading span task to measure their working memory (WM) capacity and (b) a standard cross-modal priming task to measure the lexical disambiguation time. The results revealed that (1) the underlying mechanism of the disambiguation process seemed favorable for an inhibition approach and (2) the frequency of the individual meanings of the ambiguous words and the numbers of their meanings might interact with the WM capacity during lexical access, particularly for the low-WM span group.

## Introduction

Working memory (WM) is a resource-limited storehouse in our cognitive system that is responsible for maintaining and manipulating information in an effective and efficient way in order to perform cognitive processing [1–3]. A major theoretical issue in the study of psycholinguistics relates to the nature of how working memory functions in language processing. It is well-documented that there is a strong relationship between working memory (WM) and language processing [4–10]. Past research reported that people with high WM capacity were more efficient in processing complex sentences (e.g. post-embedded part in Dutch at sentence final verb [11]) and in resolving syntactic ambiguity (e.g. relative clauses) than people with low WM capacity [12]. In the bilingual research, there were also evidence indicating that different working memory capacity would impact more on how the highly proficient L2 learners process ambiguous sentence structure than the linguistic features of their L1 [13].

However, there are different viewpoints regarding how the underlying mechanism links the working memory and language processing in our cognitive system. Some evidence supports the idea that the mechanism relating WM to language processing should follow an activation approach [14]; people with high working memory spans are more efficient and outperformed those with low working memory spans in parsing because the working memory can help people activate all relevant information supporting the language processing and sentence comprehension [10,12]. However, other evidence has been put forward for an inhibition approach [15] to relate WM and language processing, that people with high working memories can suppress and inhibit all irrelevant information effectively during language processing. Hence, we can see the importance of this theoretical issue because these two opposite approaches may lead to totally different interpretations to the underlying psychological mechanism between working memory and language processing [16].

The issue of lexical ambiguity resolution, one of the core competences of human language processing, presents an appropriate case that can reflect clearly the relationship between WM and spoken language processing [17]. In the review paper of Simpson [18], lexical ambiguity processing is that when we hear or see an ambiguous word, alternative meanings associated with the lexical item will be activated simultaneously and context effects can resolve the ambiguity by selecting the most appropriate meaning at different time points during lexical access. For example, *park* means a recreational place; this is the dominant meaning and a noun. However, it can also mean an action for parking a vehicle, which has another meaning and is a verb. Hence, when the word *park* is encountered, listeners may be faced with the ambiguity and need to resolve the appropriate sense of the word to comprehend the sentence. Finally, listeners can choose the most appropriate meaning of the ambiguous word based on the contextual cue in the ongoing sentence. Therefore, an explanation of what listeners do to resolve the ambiguity when they hear a disambiguation cue (or contextual cue) can help to clarify the nature of the connection between WM and language processing. In the literature on lexical ambiguity research, the exhaustive access hypothesis (in contrast with the context-dependency hypothesis [19,20]) claimed that all the associated meanings of an ambiguous word will be activated shortly following the occurrence of the word, and the context (or disambiguation cue in the context) can help to select the contextually-appropriate meaning at a late post-access stage, at least after the duration of four-syllable [21,22]. Therefore, when a listener hears a semantically-neutral sentence, all the associated meanings of the ambiguous word will be automatically activated initially until a disambiguation cue is present.

Past studies of lexical ambiguity resolution [17] have paid relatively less emphasis on the role of working memory played in the processing, which leaves an open question about the specific mechanism underlying participants’ disambiguation processes. For example, under the framework of WM is a capacity-constrained model [14], the simultaneous activation of all meanings associated with the ambiguous word does not necessarily delay the disambiguation process due to multiple access. It may mean, instead, that they do not have sufficient working memory resources to inhibit irrelevant meanings during the disambiguation process. To discriminate clearly between the two contrasting approaches underlying how WM supports language processing, the role of the working memory needs to be considered seriously [23]. There are various definitions of working memory used by different researchers on different topics [24]: Computer WM [25], Life-planning WM [26], Multicomponent WM [1–3], Recent-event WM [27], Storage-and-processing WM [28], Generic WM [29], Long-term WM [30], Attention-control WM [31], and Inclusive WM [32]. The different definitions are sometimes used to support the specific discipline the researchers worked on and not exclusively in the psychological research due to its malleable nature. And, we conceptualized working memory as a combinational storage-and-processing unit [28], in contrast to the usage as a multicomponent WM here. Because language comprehension involves continuous cognitive integrational processes between incoming stimuli and learnt knowledge in our mind. Therefore, it fits to the definition of WM as a temporary capacity-limited space that can combine the information storage and processing together.

In English studies, with the control of working memory, evidence shows that activation and inhibitory processes are both at work in lexical ambiguity resolution. Miyake, Just, and Carpenter [14] tested participants with a context in which the disambiguation cue was presented eight words after the ambiguous words. In this case, the contribution of working memory tended to be related to maintaining multiple meanings of words until the readers encountered the cue. Gunter, Wagner, and Friederici [11] observed, in their ERP data, that high-span participants showed a smaller *N400* in the dominant disambiguation condition than in the subordinate one, while low-span participants were the same in the two conditions. In these cases, working memory was found to be associated with inhibiting irrelevant information efficiently and this, in turn, smoothed the comprehension process. Therefore, the authors proposed that the inhibitory processes were more likely underlying working memory used during sentence processing. Furthermore, Gadsby, Arnott, and Copland [33] tested participants using homographs (words with identical orthographic form but distinct meanings) in a priming task. They found that those with high working memory spans exhibited a pattern of priming for congruent conditions and inhibition for incongruent conditions. The performance of participants with low working memories was also modulated by the frequency of meaning. With regard to dominant meanings, these participants showed inferior inhibitory processes. This indicated that working memory influences inhibitory processes in lexical ambiguity resolution. Overall, these results indicated that the role of working memory can function in different ways in English reading, either activation or inhibition, which also depends partly upon the frequency of individual meanings [34].

In the present study, we made use of Cantonese homophones (a very ambiguous and highly context-dependent language [35]) as the testing materials to examine this important issue. Approximately 10,000 Chinese characters are commonly used in Cantonese Chinese while the total number of syllables in Cantonese is only about 1700 [36]. For example, a Cantonese monosyllable /si^1^/ has up to 14 meanings (e.g., teacher, lion, silk, corpse, poem, private, think, etc.). Upon hearing the syllable / si^1^ / in a neutral sentence (or alone), native Cantonese listeners might or might not be activated all 14 meanings of the single syllable initially until they hear the disambiguation cue in the sentence [37,38]. Therefore, Cantonese monosyllabic homophone (words with identical pronunciation but distinct meanings) is highly ambiguous on a lexical-morphemic level. Certainly, some may argue that native Cantonese speakers will use disyllabic words (more than a single syllable) to clear up the alternative meanings so as to avoid confused interpretations, but it is not uncommon to use a single syllable in their daily conversations; furthermore, to be consistent with the relevant Cantonese studies on this lexical ambiguity topic, we chose to use monosyllabic homophone as the testing materials here. In addition, sentence processing in spoken domain requires a higher cognitive load and hence we used homophones (cf. homographs) as the testing target in the present study in order to distinguish clearly how the two approaches of working memory functioned in language processing. In the literature on lexical ambiguity resolution in Chinese language, large amount of evidence supported the context-dependency hypothesis [37–41]. Those results came from a series of experimental studies on this topic, using different experimental designs, tasks and measurements (such as behavioral reaction time and eye-movement data).

Following the rationales of the two different views about the linkage between WM and language processing, we set two hypotheses to examine the mechanism underlying the relationship between WM and language processing, using the homophone processing as the testing case. We illustrate them in the following example.

First, listeners are invited to hear the following sentence and then ask to name aloud the visual probe (semantically-related to one type of the disambiguation cue) that is presented on the computer screen. The response latencies will be calculated from the onset of the visual probe.

Spoken sentence: “You can buy the **BAT** from a very famous, but nearby (a) sport shop / (b) pet shop / (c) hotdog shop over there”.

Visual probes: (i) athlete (ii) animal and (iii) snack.

#BAT is the target homophone; (a) and (b) are disambiguation cues related to the dominant and subordinate meanings of the homophone respectively and (c) is the unrelated control.

If the underlying mechanism connects WM and language processing is in an activation approach (i.e., high-WM span listeners will activate all meanings of the homophone while the low-WM span listeners can activate one meaning only), then it is predicted that, for the high-WM span listeners, the response latencies for (i) should be equal to (ii) and both (i) and (ii) should be faster than (iii); whereas for the low-WM span listeners, the response latencies for (i) should be the fastest, followed by (ii) and then (iii). Because the high-WM span listeners can activate and maintain the two meanings associated with the homophone until they encounter the disambiguation cue (to select the contextually appropriate meaning) later on; while the low-WM span listeners can only activate and sustain the activation of the only one meaning associated with the homophone until they encounter the disambiguation cue due to the limited resource.

On the contrary, if the underlying mechanism connects WM and language processing is in an inhibition approach (i.e., high-WM span listeners can inhibit irrelevant meanings of the homophone rapidly after the occurrence of the disambiguation cue while the low-WM span listeners cannot efficiently inhibit irrelevant meaning even after the occurrence of the disambiguation cue). Then different predictions will be made: for the high-WM span listeners, the response latencies for (i) should be faster than (ii) and then (iii), whereas for the low-WM span listeners, the response latencies for (i) should be equal to (ii) and probe types (i) and (ii) should both be faster than (iii). Because the high-WM span listeners can quickly suppress (one of) the irrelevant meanings associated with the homophone when they hear the disambiguation cue (only the contextually appropriate meaning remains); while the low-WM span listeners cannot (quickly) suppress the irrelevant meanings associated with the homophone when they hear the disambiguation cue (two alternative meanings remain) due to the inadequate resource to do inhibition efficiently.

In this study, we attempted to examine the abovementioned predictions by a cross-modal priming task. Before that, we also conducted a reading span task with all participants so as to assess their working memory capacities for assigning them to high-WM span and low-WM span groups [11,13]. The results should provide new and clear evidence to clarify the underlying connection between working memory and lexical ambiguity resolution in particular and language processing in general.

## Experiment

### Method

#### Participants

Sixty native Cantonese speakers (Male: 18 and Female: 42, mean age: 23.6) were recruited to participate in the study. They were all undergraduates from Hong Kong with no vision, speech or hearing deficits. They were invited to complete a Language History Questionnaire, LHQ2.0 [42] to record their demographical information, language experience and language proficiency and then did two experimental tasks (a reading span task and a cross-modal priming task) one by one. They were paid HK$50 for their participation. Written informed consent was obtained from each participant and they were all informed verbally about the procedure. The study was approved by the HREC of the Education University of Hong Kong.

#### Task one: Reading span task

We adapted the standard reading span task [28] to assess the participants’ working memory capacities. During this task, the participants were asked to read aloud a series of 15–17 Cantonese words sentences (from two to eight sentences, see Appendix I in S1 File) presented on the computer screen one by one, and at the same time to remember the last word (i.e., the answer) of each sentence sequentially. Their task was to write down the answers on a record sheet in correct order and the task would be stopped when the participants made consecutive errors on the final word recall. Following the previous scoring method of the task [43], the full score was 20. The duration of this task was about 5 minutes.

#### Task two: Cross-modal priming task

We used a standard cross-modal priming task to evaluate the lexical ambiguation process of Cantonese [37,38,41].

*Materials*. Thirty Cantonese homophones were selected (see Appendix II in S1 File), each with two different meanings (i.e. dominant and subordinate). The information about homophone frequency for each meaning was based on previous works of Cheung & Flynn [44] and Ho and Jiang [45] (and the associated database). Thirty semantically-neutral sentences (see Appendix III in S1 File for sample sentences) were constructed for each homophone. Each homophone was then located at the third or fourth position in the semantically-neutral sentence and the disambiguation cue were located at the twelfth or later position in the same sentence so as to assess the sustainability of the activated meaning(s) [15,41,46].

*Norming experiment (sentences and visual probes)*. Two groups of 20 native Cantonese speakers were recruited to judge the relationship between the sentence context and the disambiguation cue. The first group of 20 native Cantonese speakers was given all the thirty test sentences without the disambiguation cue, and they were asked to complete the sentences as well as to rate the naturalness of the sentences. Across the twenty raters, the mean rating was 3.1 following the method used in Marslen-Wilson and Welsh’s study [47] and this result proved that the test sentences were all semantically-neutral or ambiguous. Moreover, all raters agreed that all the testing sentences read natural in Cantonese speech. The second group of another 20 Cantonese speakers was asked to propose the appropriate disambiguation cues to each meaning of the homophones. Using a simple semantic relatedness norm task, we asked that 20 participants to think of three Chinese words that closely matched with the two meanings of each homophone and we selected the most frequently proposed words as the disambiguation cues for the two meanings of each homophone.

In addition to the sentence context and disambiguation cues, we selected the appropriate visual probes (Chinese characters) based on the results of a semantic relatedness norm experiment conducted via the Internet by recruiting another separate group of one hundred native Cantonese speakers. In that experiment, the participants were asked to think immediately of three Chinese words with the same or a closely related meaning to each disambiguation cue, and the mostly frequent words they listed were used to be the related visual probe for the two meanings associated with each spoken homophone. The unrelated probes were selected randomly from the same source. Therefore, the visual probes were related semantically to either (a) the dominant meaning of the spoken homophone, (b) the subordinate meaning of the homophone or (c) an unrelated control. All the visual probes in each experimental condition were matched as possible with the same category of initial phonemes, frequency information and number of strokes [44,45].

An example:

*Goh3 dui1* ***si1*** *fong3 joh2 heung2 do6 gam3 noi6 do1 mo5* *(a) hok6 saang1 / (b) choi4 fung3 / (c) hon1 gaang1* *lai4 loh2*. (嗰堆詩*/*絲放左響度咁耐都無(a)學生/ (b) 裁縫/ (c) 看更黎囉。)
(The **poem/silk** was placed in the corner for such a long time and there is still no *(a) students / (b) tailor / (c) security guard* to pick it up.)

The three visual probes in this sentence were: *foh3sat1*課室 “classroom” (related-dominant), *gaau3jin2* 鉸剪 “scissors” (related-subordinate), *jai3fuk6* 制服 “uniform” (unrelated control). The first two visual probes were the disambiguation cue of the homophone.

*Design*. The 60 participants were assigned randomly to receive an equal number of 30 sentences (with any one of the three different types of visual probes). The order of presentation for the sentences was pseudorandomly arranged such that the visual probes did not consecutively bias spoken homophones. The order of presentation was counterbalanced across all participants. No participant heard the same homophone and saw the same probe twice.

*Experimental apparatus*. The test sentences were read by a female native Cantonese speaker at a normal speech rate and recorded on a voice recorder. The spoken sentences were transformed and digitized into a computer. A computer program called PsyScope [48] controlled the presentation of the materials. A microphone which was used to register the participants’ vocal responses and naming latencies (counting from the onset of the visual probe until vocal response). A SONY tape-recorder was also used record participants’ vocal responses so as to check for their accuracy.

*Procedure*. The participants did the experiment individually in a quiet experimental room. Before the experiment, the experimenter explained the task in Cantonese. First, they were told that they would be hearing an auditory presented sentence through a speaker and then, towards the end of the sentence, they would see a Chinese character (visual probe) presented on the computer screen at the onset time of the disambiguation cue until a response was made. Participants’ task was simply to name the Chinese character aloud into the microphone as quickly and accurately as possible. Before the actual experiment began, they were given a practice session in which they heard a set of separate but similar sentences. The cross-modal priming experiment took about twenty minutes.

### Results

#### Reading span task

Out of the 60 participants, two groups each of 26 were classified as the high-WM span group (mean: 15.9) and low-WM span groups (mean: 8.3), *t*(50) = 19.35, *p* < .001, based on their performances on the reading span task. Eight participants were excluded due to their very low scores (below 5) in the reading span task.

#### Cross-modal priming task

**Fig 1:**
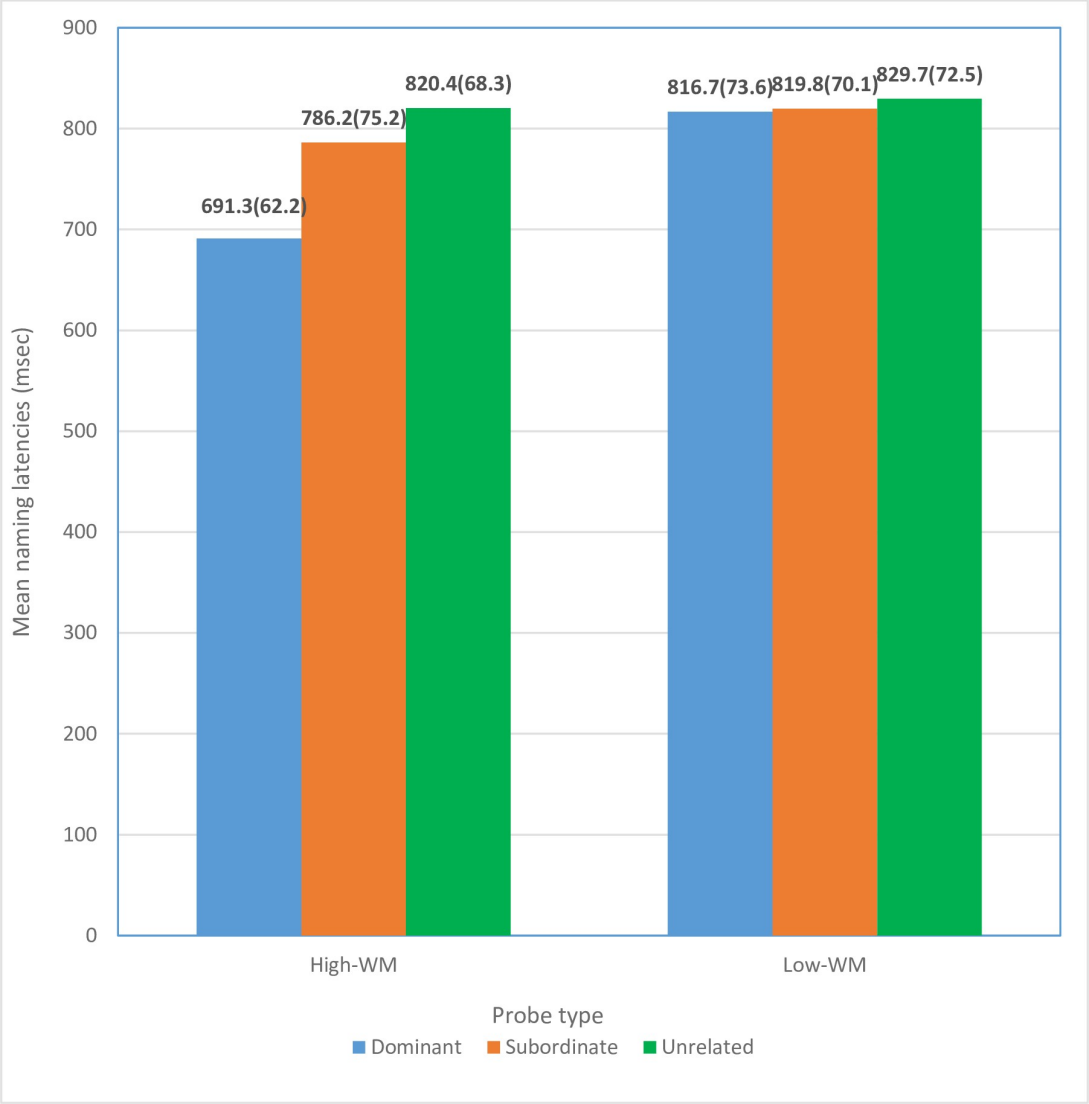
Mean naming latencies (S.D.) as a function of probe type for the two WM span groups.

The mean naming latencies as a function of probe type for the two groups (high-WM span and low-WM span) are presented in Fig 1. The response latencies were counted from the onset of the visual probe to participants’ vocal responses to allow sufficient time to process the disambiguation cue [11,41]. Errors (i.e., participant named a totally different word from the target visual probe) were very rare (approximately 0.05 on average), and therefore error data were not analyzed any further in the present study.

First, we conducted an overall 2 (between- participant variable: high vs. low WM span) x 3 (within- participant variable: types of visual probe) repeated-measure ANOVA to test the interaction between the two variables. The interaction between the two variables was significant at F(1,50) = 12.26, *p* < .05, η^2^ = 0.78, which suggesting that participants with high WM span processed the different types of visual probe differently.

To further explore the effects clearly, two repeated-measure ANOVAs were conducted on the naming latencies for the different types of visual probes for the two WM span groups.

For the high-WM span group, the results revealed a clear main effect on probe type. F(1,25) = 11.75, *p* < .05, η^2^ = 0.83. Post hoc tests (with Bonferroni’s correction) showed that a mean of 691.3 milliseconds for the naming latencies to access the dominant homophone meaning. This was faster than the time to access the subordinate homophone meaning, 786.2 milliseconds and unrelated control, 820.4 milliseconds (all *ps* < 0.05). These results were clearly consistent with the predictions generated from the inhibition approach. Similarly, for the low-WM span group, the ANOVA results revealed a main effect on the probe type, F(1,25) = 4.36, *p* < .05, η^2^ = 0.63. The mean of the naming latencies to access the dominant homophone meaning was 816.7 milliseconds, the subordinate homophone meaning was 819.8 milliseconds and the unrelated control was 829.7 milliseconds. Post hoc analyses (with Bonferroni’s correction) also confirmed that the access times for both dominant and subordinate homophone meaning were comparable (*p* > 0.05) but faster than the control (*ps* < 0.05). Together with the overall trend of result, the patterns of results were consistent with the inhibition approach, to a large extent explaining how working memory functioned in the lexical disambiguation process.

## General discussion

The present study is a first attempt to examine the role of working memory played in the lexical disambiguation process in Cantonese. We used ambiguous Cantonese words (i.e., monosyllabic homophones) as the testing item because monosyllabic homophones in Cantonese are ambiguous at a lexical-morphemic level and Cantonese is a highly context-dependent language. The question about how to resolve the lexical ambiguities in Cantonese homophones can clearly uncover the nature of the relationship between working memory and language processing because the two approaches (activation and inhibition) predicted the disambiguation processes differently. Hence, one of the main objectives of the present study was to use the findings about Cantonese homophone processing to clearly reveal the underlying mechanism of lexical ambiguity resolution and its relationship to working memory [24,28].

In this study, we firstly classified the participants into high-WM span group and low-WM span group according to a reading-span task [11,13]. The two span groups were tested further by a standard cross-modal priming task. This task was proved to be a useful paradigm to assess the various lexical and contextual effects on lexical disambiguation process in Chinese [35,37,38,41]. From the cross-modal priming task, we observed that the high-WM span listeners named the visual probes (disambiguation cue) related to the dominant meaning of the ambiguous word much faster than the probes that related to the subordinate meaning of the ambiguous word and the unrelated control. However, for the low-WM span listeners, the naming time was not differed for the two types of visual probes that were related to the dominant and subordinate meaning of the ambiguous word. The results implied that the high-WM span listeners could inhibit irrelevant meanings of the ambiguous word quickly after the occurrence of the disambiguation cue whereas the low-WM span listeners could not. This pattern of results supported the inhibition approach would be a more likely approach underlying WM and language processing but not the activation approach. Because, if the mechanism connecting WM and language processing is in an activation approach (i.e., high-WM span listeners will activate and maintain all meanings of the ambiguous word while the low-WM span listeners can activate and maintain the one (dominant) meaning only when they hear the ambiguous item), then there would not be any differences on naming times between visual probes that were related to both meanings of the ambiguous word for the high-WM span listeners as well as a faster naming time for probes related to the contextual appropriate meaning of the ambiguous word than the other probes for the low-WM span group. Nonetheless, the present pattern of results did not support these predictions from the activation theory.

Obviously, the results for both groups implied that the underlying mechanism of the disambiguation process may be favorable for an inhibition approach. The working memory can help listeners to suppress or inhibit the irrelevant meanings associated with the ambiguous word immediately when they hear the disambiguation cue, which is in line with the studies of Gernsbacher & Faust [15] and Gunter, et al. [11]. Moreover, we observed that frequency of the individual meanings of an ambiguous word and the numbers of meanings of the ambiguous words might interact with the WM capacity during lexical access, particularly for the low-WM span group. The low-WM span listeners could not inhibit the irrelevant meanings associated with the ambiguous words quickly due to the resource and capacity-constraint in WM [14,28], so they accessed the meanings of the ambiguous words in a reordered manner [49]. That maybe the alternative explanation to the present results, that is the frequency of individual meanings matters on the accessing time and this factor interacts with the WM capacity of the participants [50]. One may argue that the frequency effect on the two alternative meanings associated with the homophone can explain the differentiative effects, i.e., different degree of priming from the two homophonic meanings. Nonetheless, two important points should be noted here. First, all the ambiguous words we used in the present study have two meanings (i.e. dominant and subordinate) but frequency difference between the two meanings was slight and comparable across conditions; and second, all the ambiguous words we used in the present study have not only two meanings but most of them have more than three or four relevant meanings (though at a lesser magnitude on frequency to the third or fourth meanings). The main focus here is on the numbers of meanings associated with the homophone that our memory system can manage (how many meanings our memory system can hold and sustain as well as how many meanings the system can inhibit rapidly) but not the depth of dominance to the individual meanings (or the order of access between the alternative meanings) of the homophone. This is also the reason why we used the lexical ambiguity resolution of Cantonese homophone to rigorously examine the relationship between WM and spoken language processing [11,18,33,37,38]. Thus, the present results clearly support that the high WM-span listeners can be more flexible to resolve the lexical ambiguity due to the higher mental resource they had to undergo the inhibition process. In any case, frequency of the individual meanings of the ambiguous word as well as the number of meanings [51,52] are the other factors that we need to be investigated in future studies. Together with previous evidence, the patterns observed in the present results added new information to disentangle the two approaches underlying the strong linkage between WM and lexical disambiguation processes in Cantonese Chinese.

Finally, two important issues that need to be investigated before a more comprehensive picture of this activation-inhibition distinction issue can be drawn. Firstly, we adopted the definition of WM as a combinatory storage-and-processing system [28] instead of a multicomponent system in the present study (see Cowan [24] for a review). The sub-components of the WM system (such as executive function or others [3]) may also be essential to unfold the dynamics of the underlying linkage between WM and general language processing. Secondly, one should note that the present study mainly used Cantonese-spoken homophones to be a single testing case. Therefore, ongoing experiments are being designed and conducted in our laboratory to examine these issues further (1) by using Mandarin homophones and other type of ambiguous words (i.e., Chinese homographs); (2) using other general language processing tasks and working memory measurements to tap into differentiative effects on different sub-components of the working memory system; and (3) using different paradigms to see the fine-grained changes along the temporal structure of the spoken sentence (e.g. eye-movement technique and EEG measure, Gunter, et al. [11]; Leinenger et al. [34]).

## Supporting information

S1 File (DOCX)
