# Supplementary material for: Working memory and lexical ambiguity resolution in Cantonese Chinese
Source: PLoS One. 2021 Mar 10;16(3):e0248170. doi: 10.1371/journal.pone.0248170 (PMC7946315; doi:10.1371/journal.pone.0248170)
Supplement: S1 File — (DOCX) [file pone.0248170.s001.docx]

Appendix I: Sample Cantonese sentences used in the Reading Span task

1. **我睇過好多有關呢個研究題目**嘅論文**。**
2. **呢份係功課**嘅內容，聽日堂上要做**報告。**
3. 佢出國留學無自己去租屋住，都係住宿舍**。**
4. **我**今晚同一班好耐無見嘅同學去食火鍋**。**
5. **只要你俾心機讀書，一定可以入到大學。**

Appendix II: Spoken Cantonese Homophones used in the Experiment

baan1 班、斑 baan2 板、版

bo3 布、報 boh1 波、坡

cheung1 槍、窗 cheung4 牆、場

chi4 池、詞 do2 島、賭

foh3 貨、課 gau2 狗、九

jeung3 賬、帳 jeung6 象、杖

kei4 旗、期 kwan4 裙、群

ma5 馬、碼 min6 面、麵

mo6 霧、墓 ping4 瓶、坪

saam1 衫、三 sau2 手、艘

seung1 箱、霜 si1 詩、絲

sin3 線、扇 sui3 稅、歲

tong4 糖、堂 wa2 話、畫

wan4 雲、魂 woh1 窩、鍋

woo4 湖、狐 yuen4 圓、猿

*Note: The number of each syllable represents the lexical tone

Appendix III: Sample sentences (visual probes) used in the Experiment

**布**、**報**

嗰啲布/報今日會送嚟，收到嘅話就派比裁縫/學生/律師啦。

衣服 (DOM) - 書本 (SUB) - 天橋 (CON)

The **cloth/newspaper** will be delivered today and please send them directly to the **tailor/students/lawyers** once received.

**槍**、**窗**

我一開槍/窗就聽到外面啲雀仔驚慌/唱歌/工作嘅叫聲。

子彈 (DOM) - 門口 (SUB) - 女士 (CON)

When I fired/opened the **pistol/window**, I heard the **terrifying/singing/working** sound screening from the birds outside.

**池**、**詞**

呢個池/詞好似好耐都無見過有漁民/老師/紙巾用啦。

水塘 (DOM) - 課本 (SUB) - 時鐘 (CON)

This **pond/word** seems not to be used by **fishermen/teachers/tissues** for a long time.

**旗、期**

呢啲旗/期一定要好好利用，我哋可以喺頒獎禮/放長假/食火鍋時用到。

信號 (DOM) - 時間 (SUB) - 山羊 (CON)

This **flag/period of time** should be utilized in a better way and we can use it in the **prize-ceremony/holiday/hotpot** time.

**話、畫**

呢啲話/畫真係好怪，我估一定要語言學家/藝術家/運動員先識。

文字 (DOM) - 色彩 (SUB) - 年歲 (CON)

That **language/painting** seems weird and I think that only **linguists/artists/athletes** can understand.

**詩、絲**

嗰堆詩/絲放左響度咁耐都無學生/裁縫/看更黎攞。

課室 (DOM) - 鉸剪 (SUB) - 制服 (CON)

The **poem/silk** was placed in the corner for such a long time and there is still no *(a) students / (b) tailor / (c) security guard* came to pick it up.

**線**、**扇**

呢啲線/扇非常實用，我認為最適合用嚟繡花/撥涼/旅遊。

冷針 (DOM) -涼風 (SUB) - 淋浴 (CON)

This **thread/fan** is very useful, and I can use it for **embroidery/cooling/travel**.

**湖**、**弧**

我對湖/狐嘅概念好有興趣，所以我會選修同地質學/動物學/血液學相關嘅科目。

海洋 (DOM) – 豺狼 (SUB) - 飯堂 (CON)

I am quite interest to the concept of **lake/fox**, so I will take the elective course from **geology/zoology/hematology**.
